# Influence of microbial biomass content on biodegradation and mechanical properties of poly(3-hydroxybutyrate) composites

**DOI:** 10.1007/s10532-023-10038-1

**Published:** 2023-07-04

**Authors:** Felix Eckel, Korbinian Sinzinger, Daniel Van Opdenbosch, Doris Schieder, Volker Sieber, Cordt Zollfrank

**Affiliations:** 1 Chair for Biogenic Polymers, TUM Campus Straubing for Biotechnology and Sustainability, Technical University of Munich, Schulgasse 16, Straubing, 94315 Germany; 2 Chair for Chemistry of Biogenic Resources, TUM Campus Straubing for Biotechnology and Sustainability, Technical University of Munich, Schulgasse 16, Straubing, 94315 Germany

**Keywords:** Polyhydroxybutyrate, Biodegradation, Biostimulation, CO_2_-evolution, Molecular weight, Microbial biomass

## Abstract

Biodegradation rates and mechanical properties of poly(3-hydroxybutyrate) (PHB) composites with green algae and cyanobacteria were investigated for the first time. To the authors knowledge, the addition of microbial biomass led to the biggest observed effect on biodegradation so far. The composites with microbial biomass showed an acceleration of the biodegradation rate and a higher cumulative biodegradation within 132 days compared to PHB or the biomass alone. In order to determine the causes for the faster biodegradation, the molecular weight, the crystallinity, the water uptake, the microbial biomass composition and scanning electron microscope images were assessed. The molecular weight of the PHB in the composites was lower than that of pure PHB while the crystallinity and microbial biomass composition were the same for all samples. A direct correlation of water uptake and crystallinity with biodegradation rate could not be observed. While the degradation of molecular weight of PHB during sample preparation contributed to the improvement of biodegradation, the main reason was attributed to biostimulation by the added biomass. The resulting enhancement of the biodegradation rate appears to be unique in the field of polymer biodegradation. The tensile strength was lowered, elongation at break remained constant and Young’s modulus was increased compared to pure PHB.

## Introduction

Biodegradable polymers are part of the solution to environmental problems caused by waste and by the accumulation of microplastic (Agarwal 2020). By composting, they may also simplify the process and costs of plastic waste disposal (Rujnić-Sokele and Pilipović 2017). In many cases the biodegradation is still too slow or varies between different environments. Therefore, tailoring the biodegradation and increasing the biodegradation rate is desirable. This is not only true for the disposal of plastic waste, but also for different applications like mulch foils or fishing nets, where the stability and functionality of the product has to be balanced with its biodegradation (Kyrikou and Briassoulis 2007).

Additives may influence the biodegradation process by altering material properties such as the crystallinity of the matrix polymer, the water uptake or the surface area before and after biodegradation of the additive. Other important parameters for the biodegradation rate are the molecular weight of the components, the microbiome and its activity, as well as environmental factors (Chandra 1998; Tokiwa et al. 2009).

Some additives may have an effect on the material properties and also on the microbial activity. An increased microbial activity by the addition of compounds which provide nutritional value and can be easily metabolized, is called biostimulation (Tyagi et al. 2011).

Many microorganisms can digest the polymer itself or its hydrolytic degradation products. After the consumption of readily available carbon sources, the microbiome has the ability to make use of another carbon source, for example the biodegradable polymer (Harder and Dijkhuizen 1982). One limiting factor for the biodegradation of organic samples may be the competition for inorganic substrates. If there are not sufficient nutrients available, the polymer may not be biodegraded (Steffensen and Alexander 1995). A comparable problem has been studied for decades regarding the bioremedation of oil spills. Atlas et al. stated in their review that the biodegradation of hydrocarbons depends on the availability of N and P and may be limited or stimulated by scarcity or addition of available nutrients (Atlas 1981). Organic material like plant fibres have been used as an additive for PHB composites in order to improve their properties. An influence on the biodegradation could only be observed in the range of the added fraction and no biostimulation effect was observed so far (Fernandes et al. 2020) Using microbial biomass as additional substrate on the other hand may provide all the compounds which are needed for a thriving microbiome and an improved biodegradation even for secondary substrates like the polymer matrix. Therefore, microbial biomass containing many different nutrients like carbohydrates, lipids and proteins may be a possible trigger for the biostimulation of the biodegradation process and could be used by the microbiome either directly or after degradation to smaller subunits like sugars, amino acids or fatty acids. One study was published during our proceeding experiments. They analysed the potential of algae for the acceleration of PLA degradation by providing a nitrogen source for microbial growth (Kalita et al. 2021). Faster hydrolysis led to a very low molecular weight of the PLA-algae composite and therefore to an improved biodegradation.

Algae are a common kind of microbial biomass. The term “algae” is commonly used for a wide range of water organisms, ranging from bacteria to multicellular macroalgaes. Green algae, red algae and brown algae are all eukaryotes while cyanobacteria, known as “blue-green algae”, are prokaryotes (Sahoo and Seckbach 2015). They are a biogenic resource, which is available in huge quantities and shows a similar mechanical behaviour in different polymer composites: Different kinds of incorporated algae have been reported for polyethylene, polypropylene, poly(vinyl chloride), poly(vinyl alcohol), poly(lactic acid), poly(butylene adipate-co-terephthalate), poly-($\varepsilon$-caprolactone), poly(butylene succinate), isocyanate based foams and polyhydroxybuyrate (Barghini et al. 2010; Bulota and Budtova 2015; Zhang(b) et al. 2000; Zhang(a) et al. 2000; Otsuki et al. 2004; Zhang et al. 1999; Chiellini et al. 2008; Constante and Pillay 2017; Sim et al. 2010; Stoudt 2017; Torres et al. 2015; Johnson and Shivkumar 2004; Lee et al. 2008). The addition of biomass often leads to a higher Young’s modulus and lower tensile strength and elongation at break (Bulota and Budtova 2016, 2015; Chiellini et al. 2008; Torres et al. 2015). Another use for the incorporation of biomass in fossil fuel based plastic materials is the carbon storage of biologically fixed CO_2_ (Zhang(b) et al. 2000).

We chose PHB as a matrix polymer because it is biodegradable in aqueous and soil environments (Mukai et al. 1994; Volova et al. 2011; Nishida and Tokiwa 1993). It is used as a storage molecule by microbes and can be used as the only available carbon source by some microbes (Martínez-Tobón et al. 2018). Therefore, it is readily biodegradable even for higher molecular weights. One study found for example 695 strains of PHB degrading microorganisms from five different environments containing soils, compost, fresh water, marine water and sludge (Mergaert and Swings 1996). Influences on biodegradation rates are expected to be easily detectable compared to other less biodegradable polymers. So far, a similar idea with a different methodology has only been studied in a non-peer-reviewed, self-published undergraduate honors project with interesting but inconclusive results on the biodegradation of PHB-algae composites in seawater and compost where the biomass led to a slightly improved biodegradation (Stoudt 2017). Other environments like soil at ambient temperature, the use of different strains with known biomass compositions for PHB composites have not been studied so far.

We assumed, that without any interaction, the biodegradation rate of a composite can be expected to be calculated as the linear addition of the mass fractions of the components multiplied with the respective biodegradation rates. A higher biodegradation rate is therefore a sign of a positive interaction between the components.

The addition of biomass can lead to different biodegradation rates by different means. The molecular weight of the PHB matrix was expected to change by the addition of microbial biomass since the processing may lead to a higher thermal degradation and hydrolysis rates may be influenced. Lower molecular weight polymers are faster biodegradable (Kunioka and Doi 1990; Hoffmann et al. 1994; Tokiwa et al. 2009). Another factor to consider is a potential influence on crystallinity which could be expected since Barghini et al. have also observed a lower crystallinity of polyhydroxybutyrate (PHB) after addition of marine seaweed, *Ulva armoricana* (Barghini et al. 2010). Microbial biomass may also lead to a higher surface area of the polymer matrix and therefore to a higher biodegradability (Altaee et al. 2016; Meereboer et al. 2020).

A further factor is, that a higher water uptake of the composite compared to the PHB without additives may have an influence on the biodegradation rates by swelling of the samples. Hydrolysis rates shouldn’t be affected, since diffusion of water is assumed to be much faster than the hydrolysis (Antheunis et al. 2009; Meereboer et al. 2020).

Eventually, the microbial biomass composition may vary regarding fractions of compounds and elemental composition. Depending on the nutritional needs of the microbiome, these differences may lead also to different biodegradation rates by a previously mentioned biostimulation effect.

In this study, we investigated the influence of microbial biomass on the biodegradation of PHB-composites in a simple binary system of pure microbial PHB with 100 mg/g microbial biomass in a soil environment. For this, one spherical green algae, *Chlorella sorokiniana*, and two cyanobacteria, *Synechocystis *sp. and *Cylindrospermum alatosporum,* were used. Cells of *Synechocystis *sp. were aggregated in big flakes whereas *Cylindrospermum alatosporum *consisted only of short chains of cylindrical cells.

Important factors for biodegradation were analysed, including the determination of molecular weight and crystallinity of the matrix polymer PHB as well as the composition of the microbial biomass. The composites were investigated for their water uptake, their structural characteristics by scanning electron microscopy (SEM) and their mechanical properties. The biodegradation was investigated by quantification of the evolved CO_2_ during mineralisation of the samples.

## Methods

### Materials

Bacterial poly(3-hydroxybutyrate) (PHB) powder (Biomer, Schwalbach, Germany) was used after drying in a desiccator. The autotrophic cultivated green algae *Chlorella sorokiniana* and the cyanobacteria *Synechocystis *sp. and *Cylindrospermum alatosporum* were obtained as spray-dried specimens (Algatech, Trebon, Czech Republik). They were stored at -20 ^∘^C and further dried in a desiccator before use.

### Preparation of tensile bars

Tensile bars of type 1BA according to DIN EN ISO 527 were prepared for the analysis of mechanical properties and for the use in the biodegradation experiment as well as further analysis. Samples were compounded by a HAAKE Minilab II microcompounder (Thermo Fisher Scientific, Waltham, Waltham, Massachusetts, United States of America) with co-rotating conical twin-screws at 50 rpm for 12 minutes and subsequent injection moulding (Haake MiniJet Pro, Thermo Fisher Scientific)  (DIN EN ISO 527-2 2012). Composites consisted of PHB with 100 mg/g of the microbial biomass. Compounding temperatures are listed in Table 1. The melt was heated to the compounding temperature and pressed with 400 bar for 10 s and then with 250 bar for 5 s into the mold cavity, which was tempered to 40 ^∘^C. The processing temperature of PHB and its composites differed slightly, because the temperature range where the viscosity of the melt was processable was very small and no single temperature was found to be usable with pure PHB and its composites.

**Table 1:** Compounding and injection molding temperatures are reported for all prepared materials

Material	Temperature [^∘^C]
PHB	180
PHB*-Synechocystis*	177
PHB-*Cylindrospermum*	177
PHB*-Chlorella*	177

### Tensile testing

The mechanical properties were determined with a universal tensile testing machine (smarTens 010, Karg Industrietechnik, Krailling, Germany) on at least 8 specimens. The elongation was recorded with contact displacement transducers. Force was applied from start with 1 mm/min until a initial load of 1 MPa allowed the settling of any effects caused by tensile bar mounting. After 1 MPa was reached, samples were pulled with 5 MPa/min until an elongation of 0.5  % and finally 10 mm/min were applied until fracture occurred. Tensile strength was determined as the maxima of the stress-strain-curve, Young’s moduli were evaluated at the elastic deformation region and elongation at break was determined when the measured stress fell below 75 % of the recorded tensile strength. Work of fracture was determined as the integral of the stress-strain curve. The tensile bars were then analysed and later used for the biodegradation tests.

### Scanning electron microscopy

We examined the fracture planes of the tensile bars in order to compare possible influences of the microbial biomass distribution and matrix cohesion on differences of mechanical properties and the biodegradation of the composites. After tensile testing they were sputtered with gold and examined with a scanning electron microscope (DSM940, Carl Zeiss, Oberkochen, Germany). The accelerating potential was set to 5 kV.

### Crystallinity

Differences of the crystalline fraction of the tensile bars may lead to different biodegradation rates. Therefore, the crystallinity of the tensile bars was determined by X-ray diffractometry (MiniFlex 600, Rigaku, Tokyo, Japan) with a copper anode and a silicium strip detector (D/teX Ultra, Rigaku) from 10^∘^ to 80^∘^. Measurements were executed in 0.02^∘^ steps with 5^∘^ per minute. For each composite, one measurement of the top, middle and bottom part of one tensile bar were taken and averaged. The crystallinities were calculated after Rietveld-refinement with the software BGMN (version 4.2.23) and according to Ruland and Vonk as published elsewhere (Ruland 1961; Vonk 1973; Doebelin and Kleeberg 2015). Atomic coordinates of the PHB $\alpha$-form were taken from Wang et al. (Wang and Tashiro (2016).

### Molecular weight

Molecular weight of the samples were analysed since it is one of the most important factors affecting biodegradation and mechanical properties. 450 to 500 mg of the mixed samples for the biodegradation test were dissolved in chloroform at a concentration of 5 g/L for 45 min under reflux. Molecular weight was determined by size exclusion chromatography (SECcurity GPC System, PSS, Mainz, Germany) with a flow of 0.7 mL/min, a set of SDV 5 µm columns containing a precolumn, a 100,000 Å column, a 1000 Å column and a refractive index detector (1260 Infinity, Agilent, United States of America). Polystyrene standards from 3250 g/mol to 3.2$\cdot$10^6^ g/mol were used for calibration.

### Microbial biomass composition

Differences in biomass composition may influence the properties and biodegradation of the respective PHB composites. Therefore, total solids, ash content and the contents of starch, protein, lipids and structural saccharides were analysed according to the standard laboratory procedures (LAPs) as described by the national renewable energy laboratory (Laurens 2013) and reported by us before (Sinzinger et al. 2022).

### Biodegradation test

Aerobic biodegradation of organic material leads to the conversion to CO_2_, biomass and water. The evolving CO_2_ is therefore a good measure for biodegradation (Müller 2005). We chose a soil with a loose texture in order to allow the aerobic biodegradation to take place without anaerobic spots. Loamy humous soil was acquired from the local composting facility (Zweckverband Abfallwirtschaft Straubing Stadt und Land, Straubing, Germany) containing 50 % compost and 50 % topsoil. The soil was sieved through a 1 mm mesh to remove organic waste residues. The water content was raised to improve biodegradation conditions. Muddy soil was avoided since it may prohibit aerobic biodegradation and the proper release of CO_2_. Therefore, water content was finally set to 0.3 g/g. 300 g soil were placed in each 3-L jar. The evolved CO_2_ from the soil was absorbed in 20 mL of a 1 mol/L KOH solution, which was hung in the glass without soil contact.

Evolving CO_2_ from soil without samples was analysed to ensure that all jars were emitting equal amounts of CO_2_. Four jars containing only soil were used as blanks, three jars as a positive controls with cellulose powder (alpha-cellulose, Sigma-Aldrich, St. Louis, Missouri, United States of America) and three jars for a negative control with polypropylene. For each material, three jars were used. Tensile bars were embrittled in liquid nitrogen and shattered with a kitchen mixer (Silvercrest, Lidl Stiftung, Neckarsulm, Germany) and sieved through a 2 mm mesh. 3 g of sample were added to each jar and then mixed thoroughly with the soil. Fresh KOH-solution was added to every jar and titrated every 3 - 22 days, when the pH approached 9 to ensure that the CO_2_-absorption remained quantitatively valid. The samples were titrated over the course of 132 days at room temperature.

The absorbed CO_2_ corresponds to the volume of HCl between the two inflection points of the pH-titration curve at a pH of 8.1 and 3.9. The calculated mean weight of CO_2_ of all blanks was subtracted from every jar. The carbon contents of the materials were determined in triplicate by elemental analysis (Euro EA Elemental Analyzer, Euro Vector S.P.A., Italy) and the percent biodegradation was calculated by dividing the evolved CO_2_ from each sample by the theoretical possible amount of CO_2_-evolution after complete biodegradation.

### Water uptake

Water uptake of composite tensile bars was tracked in triplicate over 95 days of immersion in demineralized H_2_O by comparing dried weight to wet weight after removal of excess water at the surface with a paper towel.

## Results

### Biodegradation of PHB composites

**Fig. 1:**
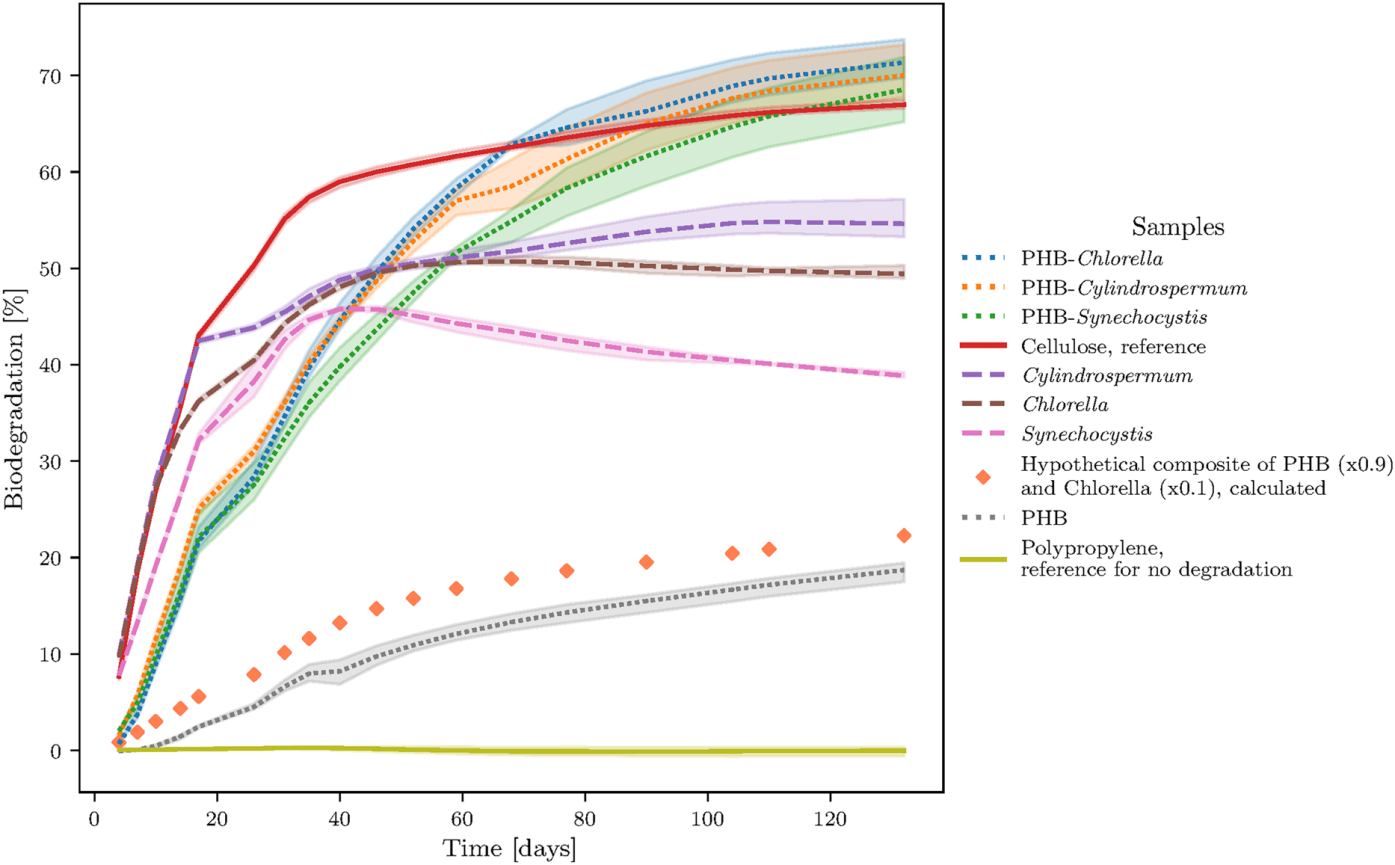
Cumulative biodegradation of microbial biomass (dashed lines), PHB and its composites (dotted lines) and the controls (solid lines). The hypothetical composite of PHB and *Chlorella*, calculated as mass fraction weighted linear combination, is shown for comparison with orange diamond markers without a line

In the biodegradation test, the completely mineralized carbon content of the samples were quantified as CO_2_. After the first 4 days, the CO_2_-evolution of the composites, cellulose and pure PHB accelerated, Fig. 1. In the days following the lag phase, the biodegradation rates of the microbial biomass were nearly as fast as cellulose and also faster than the PHB composites. After day 26, the biodegradation rates of microbial biomass and cellulose were lower than those of the composites, which then degraded faster and to a higher cumulative value. The different biomass showed a similar biodegradation pattern and reached a limit of a cumulative biodegradation which is lower than those of the composites. While the biodegradation of microbial biomass reached a limit within less than 110 days, the degradation of PHB and its composites was still ongoing when the experiment was stopped. The PHB-*Cylindrospermum*, PHB-*Chlorella* and PHB-*Cylindrospermum* composites reached a similar cumulative biodegradation value of about 70 % while the microbial biomass reached cumulative biodegradation values between 39 % and 55 %, Table 2.

Over the last weeks, the soil samples with *Synechocystis *sp*.* and *Chlorella sorokiniana* released less CO_2_ than the pure soil sample without any added substances. The soil samples with microbial biomass developed a distinct putrid smell during the degradation process whereas in all soil samples with PHB-microbial-biomass composites or cellulose, no smell was noticeable. Instead white, featureless worms with a size of up to 20 mm appeared. However, occurrence of such organisms could not be noticed in any other sample.

It appears that for most of the samples and for all of the materials biodegradation slows down between day 17 and day 26 and is accelerating afterwards. This may be attributed to some systematic error like an uncontrolled temperature change in the laboratory or to a two-step degradation process of the samples. Since the observation of a slower biodegradation rate is based on only one data sampling period, it may be an artefact.

### Molecular weight of the samples for biodegradation

**Table 2:** Comparison of mechanical, composite and biodegradation properties of the PHB-composites

	Composites	PHB	PHB*-Chlorella*	PHB-*Synechocystis*	PHB*-Cylindro-spermum*
Mechanical properties	Young’s modulus (MPa)	2,623±241	3,169±389	3,094±421	2,757±241
	Tensile strength (MPa)	39.1±2.6	30.8±1.6	30.1±5.2	22.7±7.0
	Elongation at break (%)	3.0±0.6	3.3±1.1	2.4±0.6	2.4±0.5
	Work of Fracture (J $\cdot$ m^-3^) $\cdot$10^3^	498.7±119.8	207.3±32.3	245.0±62.0	142.4±55.5
Composite properties	Mass average molecular weight *M*_*w*_ 10^3^$\cdot$(g/mol)	343±13	133±4	177±8	124±3
	Number average molecular weight *M*_*n*_ 10^3^$\cdot$(g/mol)	164±4	74±1	98±5	69±3
	Crystallinity (%)	52.2±5.0	54.2±1.3	54.4±2.3	54.4±3.0
	Mass fraction $\omega$ of water uptake after 29 days in water (%)	2.1±0.4	3.5±0.5	6.7±2.7	3.8±0.3
Biodegradation	Cumulative biodegradation after 132 days (%)	18.7±1.1	71.3±2.1	68.5±3.3	70.0±2.8
	Cumulative biodegradation of corresponding biomass after 132 days (%)	–	49.4±0.7	38.9±0.3	54.6±2.2

We analysed several factors in order to determine the cause for the faster biodegradation of the composites. A substantial degradation of molecular weight during the preparation of the samples was observed, which was increased by the addition of microbial biomass, Table 2. The PHB composite with *Synechocystis *sp. showed the lowest degradation of all composites during processing while the others degraded much stronger.

**Fig. 2:**
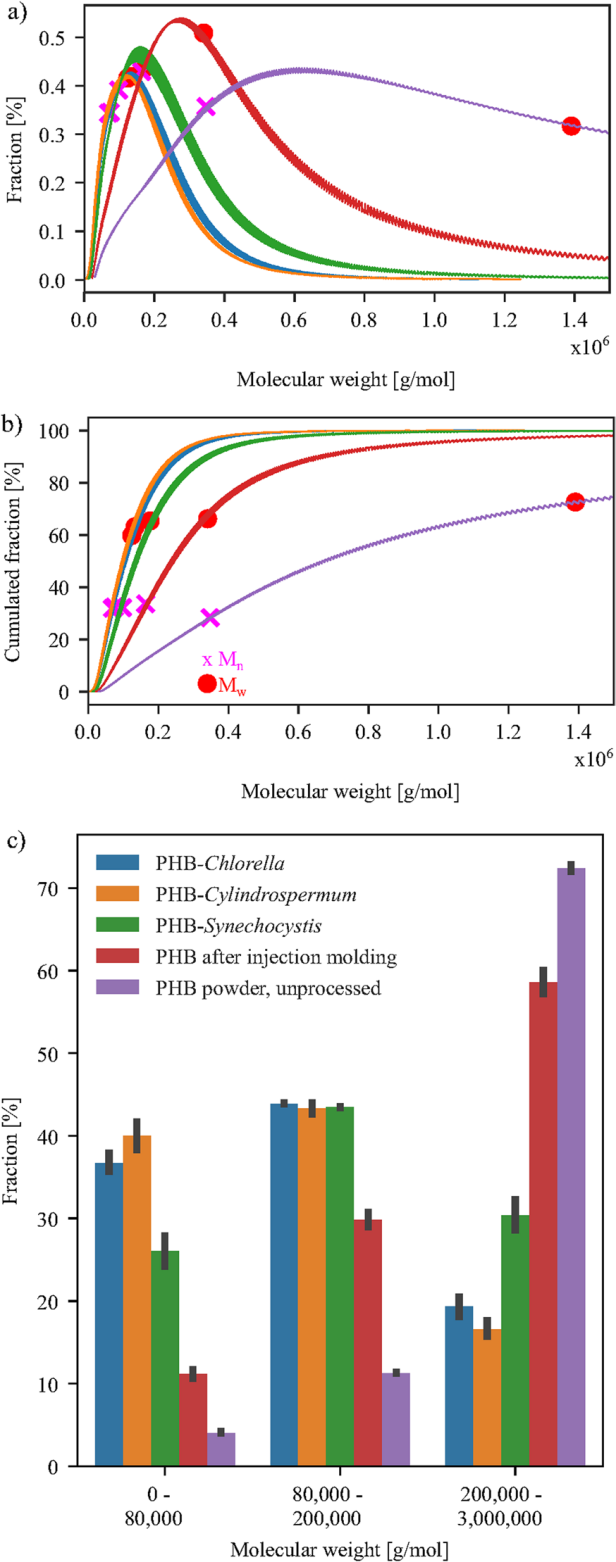
Normal **a** and cumulative **b** molecular weight distribution of PHB and its composites are shown with M_n_(pink cross) and M_w_(red dot) for each distribution. The cumulative fraction of binned molecular weight **c** of PHB is shown for all samples before the biodegradation test for the ranges from 0 g/mol to 80,000 g/mol, from 80,000 g/mol to 200,000 g/mol and from 200,000 g/mol to 3,000,000 g/mol. (Color figure online)

Pure PHB in comparison had a mass average molecular weight which was nearly twice as high as the *M*_*w*_ of the least degraded composite. The molecular weight distributions of the processed composites were more narrow than neat PHB or the unprocessed powder with a higher molecular weight, Fig. 2a). The distribution of unprocessed PHB shows values up to 10^7^ g/mol, while the processed tensile bars have polymers in a notable fraction lower than 1.5$\cdot$10^6^ g/mol. The molecular weight distributions of PHB with *Chlorella* and *Cylindrospermum* are very similar while the molecular weight distribution of PHB with *Synechocystis* is shifted to higher molecular weights.

The fractions of lower molecular weight can be more easily compared by the cumulative molecular weight distribution, Fig. 2b). To make a quantitative comparison of the molecular weight fractions possible, we have compared the fractions in 10,000 g/mol steps and aggregated them into three distinguishable bins. For each bin the fractions were similar for each 10,000 g/mol step. In Fig. 2c) the binned molecular weights of all samples in the biodegradation experiment and for comparison of the thermal degradation unprocessed PHB powder are shown. The samples show great differences in the range from 0 g/mol to 80,000 g/mol with the biomass-composites of PHB-*Chlorella*, PHB-*Cylindrospermum* and PHB-*Synechocystis* having fractions of 37 %, 40 %, 26 % respectively, while injection molded PHB has only 11 % of its mass in this range.

In the range from 80,000 g/mol to 200,000 g/mol the biomass composites exhibited equal fractions of the molecular weight from 43 to 44 %, whereas much lower fractions of 30 % were observed for PHB. In the last region, between 200,000 g/mol and 3,000,000 g/mol all samples show again large differences. The composites presented fractions of 19 %, 17 % and 30 % for PHB-*Chlorella*, PHB-*Cylindrospermum* and PHB-*Synechocystis* while most of the mass of PHB (59 %) was located in this range of the molecular weight.

### Crystallinity of PHB composites

Another determined factor is the crystallinity of the PHB samples. The crystalline fractions of the composite including the biomass are shown in Table 2. The crystalline fractions of the composites vary between 54.2±1.3 % and 54.4±3.0 % and are similar to that of pure PHB with 52.2±5.0 %. Since the values were not corrected for the added biomass, crystallinities of the polymer matrix may have slightly increased for the composites with biomass.

### Microbial biomass composition

The different biomass sources are similar in their CHNS-composition as shown in Table 3. *Chlorella sorokiniana* has a slightly lower nitrogen content, whereas *Synechocystis *sp. showed the highest sulfur content. Quantified compounds such as proteins, lipids, starch and structural saccharides occur in similar amounts in each of the microbial biomass (Table 4). The biomass consists of a mass fraction of 44 %–52 % of protein, 4 %–5 % lipids, 0 %–3 % starch and 9 %–13 % structural saccharides.

**Table 3:** Elemental composition (CHNS) as mass fraction of the used microbial biomass as determined by elemental analysis

Component	*Chlorella*	*Cylindrospermum*	*Synechocystis*
$\omega$_C_ (%)	46.52±0.26	48.02±0.12	48.43±0.33
$\omega$_H_ (%)	6.89±0.09	7.10±0.05	7.20±0.04
$\omega$_N_ (%)	6.20±0.79	10.85±0.09	10.33±0.17
$\omega$_S_ (%)	0.15±0.03	0.18±0.02	0.29±0.00

**Table 4:** Analysed mass fractions of components of the used microbial biomass as used for the tensile bars

Component	*Chlorella*	*Cylindrospermum*	*Synechocystis*
$\omega$_Protein_ (%)	44.5±0.2	52.4±0.1	48.7±0.7
$\omega$_Lipids_ (%)	4.9±0.3	4.1±0.1	4.3±0.1
$\omega$_Starch_ (%)	2.8±0.2	2.4±0.1	0.2±0.0
$\omega$_Str. sacch._^1^ (%)	8.6±0.3	8.7±0.4	12.9±0.6
$\omega$_Sum_ (%)	60.8±0.9	67.6±0.7	66.3±1.4

^1^Str. Sacch.: Structural saccharides

### Water uptake of composites

**Fig. 3:**
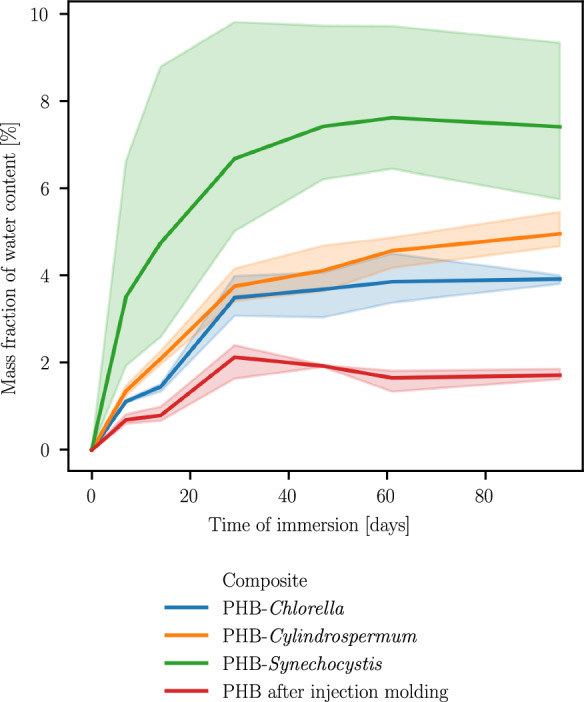
Mass fraction of water uptake of PHB and its composites over 95 days of immersion in water

Water uptake varied strongly, as can be seen in Fig. 3, even between several samples of the same material. Pure PHB showed the lowest water uptake, while composites absorbed twice as much water. PHB with 100 mg/g *Synechocystis *sp. showed an even higher water uptake than the other composites.

### Microbial biomass distribution and matrix cohesion

**Fig. 4:**
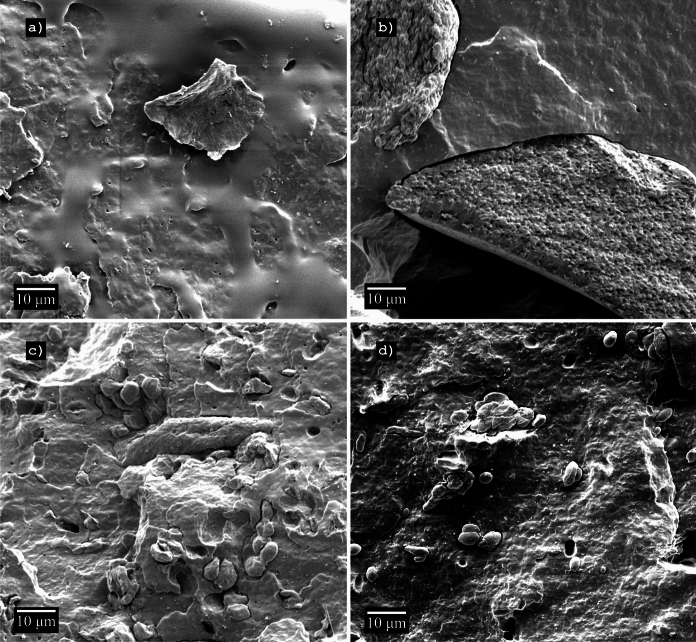
SEM images of the fracture planes after tensile testing of the PHB-composites. From top left to bottom right: **a** PHB, **b** PHB-*Synechocystis*, **c** PHB-*Chlorella*, **d** PHB-*Cylindrospermum*

SEM images were evaluated to gather information on the cell-matrix-interaction and potential differences in the surface. *Synechocystis *sp. showed rather large agglomerated tiles, whereas* Chlorella sorokiniana* was distributed more evenly and showed a good cell-matrix-interaction. The occurrence of raspberry-like aggregates was occasionally observed. Single cells of *Chlorella sorokiniana* had a spherical morphology with a diameter between 3.8 $\mu$m and 4.6 $\mu$m, whereas *Synechocystis*sp. exhibited a cylindrical morphology with diameters between 0.7 $\mu$m and 2.3 $\mu$m.* Cylindrospermum alatosporum* was distributed more homogeneously than the other microalgae, but also formed aggregates in some instances. Single cells were cylindrical with a diameter between 3.5 $\mu$m and 7.7 $\mu$m. Aggregates usually showed a lower interaction with the PHB matrix than the more finely distributed cells (Fig. 4).

### Tensile testing of the composites

The ultimate tensile strength of the composites was lower than that of the PHB, Table 2. Elongation at break of composites were similar to that of PHB, even if the values of the composites with cyanobacteria were slightly lower. The Young’s modulus of the composites increased in comparison to PHB by nearly 20 %. Work of fracture of the composites was less than half of the work of fracture of PHB. Measured differences between the composites were rather small.

## Discussion

### Interaction of components and microfauna

Our results show that PHB composites containing microbial biomass degrade faster than pure PHB without the addition of biomass. If there was no interaction between the biodegradation of PHB matrix and the added biomass, one would expect that the biodegradation of a composite can be calculated by linear addition of the biodegradation rates multiplied with the mass fraction of its components according to $P_{theo}(Composite)={\displaystyle \sum _{i}}w_{i}P_{i}$ . The calculated simulated degradation curves for non-interacting composites show, that there would be only a slight increase of the degradation according to the added mass fractions, Fig. 1. However, the measured degradation curves for the PHB-composites showed an entirely different behaviour. The composites of PHB and microbial biomass appear to biodegrade considerably faster in addition to a greater extent than microbial biomass itself or PHB alone. This suggests that the combination of PHB and microbial biomass has a strong effect on the enhancement of the biodegradation of the composite.

There are only a few studies on the effect of algae on the biodegradation of polymers. The biodegradation of PHB-algae-films investigated in compost showed mixed results. Degradation tests of PHB with 100 mg/g algae in seawater showed the highest biodegradation of 8.8 % in comparison to 2.7 % of pure PHB after 132 days (Stoudt 2017). This may be due to an improvement of the biodegradation of both components or at least one of the components. The results suggest, that the biodegradation mainly relates to the algae. One study investigated PLA-algae composites and found some improvement of biodegradation. They speculated, that the algae were used as a nitrogen source and benefited further microbial growth (Kalita et al. 2021). Since molecular weight of the PLA in the composite dropped below 10.000 g/mol, accelerated hydrolysis may have been the major reason and not a biostimulation effect as observed in our study.

The mineralization of polyvinyl alcohol (PVA) composites containing a marine green algae showed a degradation of about 45 % after 150 days. A reason for the improvement of biodegradation was not obvious due to the presence of other substances such as starch and glycerol and the lack of a neat PVA-control (Chiellini et al. 2008).

The worm-like organisms, which were observed in the jars with the cellulose reference and the composites of PHB and microbial biomass, could not be identified without doubt. They resembled nematodes or enchytraeids, but soil nematodes are typically smaller than 2 cm and the typical clitellum of enchytraeids could not be found. However, enchytraeids have been found to have a direct contribution to soil respiration of 0.3 % to 5.2 % (Didden 1993). This low number cannot explain the improvement of biodegradation in this study alone. The indirect influence might be of much greater importance. Enchytraeids and nematodes both have the ability to exert an indirect influence on the microbial activity: i) Both can influence the microbial composition, ii) they can feed on microorganisms, iii) they may disperse spores and iv) influence nutrient - especially important nitrogen - immobilization or secretion. Enchytraeids can also feed on small organic particles and microbes (Didden 1993). They have been found to inhibit fungal hyphae, which leads to a smaller fungal biomass and a higher specific respiration (Förster et al. 1995). Their influence on CO_2_ respiration depends on the soil type, the occurring species and potentially other environmental factors. Förster et al. have found that the addition of enchytraeids led to a stimulation of microbial activity, a higher CO_2_-production and their results suggests a higher amount of mineralized nitrogen was made available. Koutika et al. also observed a higher soil respiration through the addition of enchytraeids (Koutika et al. 2001). On the other hand, Van Vliet et al. could not observe an effect on CO_2_ respiration, but also a faster decomposition of organic matter and a higher nitrogen availability (van Vliet et al. 2004). John et al. also observed less CO_2_ respiration and a higher mass loss of straw (John et al. 2019).

Since we couldn’t exclude the possibility of the worms being nematodes, we will discuss also the possible effect of nematodes on biodegradation. Nematodes serve as an indicator of and also as a factor for organic substrate turnover. Microfauna, to which nematodes belong, is increasing rapidly in environments with a high organic matter concentration. Huge quantities of nematodes are a sign of rapid degradation by decomposing bacteria and accelerate the decomposition in turn. The appearance of nematodes is also influencing the microbiome composition. In the light of Griffith’s review it may seem that the microfauna is currently understudied in the field of polymer degradation and may be as important as bacteria, fungi and archaea (Griffiths 1994). Nematodes feeding on bacteria keep the decomposing bacteria in the log phase close to the maximum growth rate (Freckman 1988). A marine nematode has been shown to increase the carbon mineralisation of marine detritus up to 300 % compared to samples without nematodes (Findlay and Tenore 1982). Nitrogen mineralisation to NH_4_ and NO_3_^-^ is increased in presence of nematodes (Ferris et al. 1998). Ammonium is considered as a suitable nutrient for biostimulation and enhanced biodegradation of oil spilled soils (Tyagi et al. 2011).

Therefore, observed improvement of biodegradation seems to be the result of a biostimulation effect. Easily biodegradable organic material like cellulose or the combination of the PHB and microbial biomass triggered the microfauna and led to a higher microbial activity and subsequent higher biodegradation by the above mentioned mechanisms.

The lower CO_2_-evolution of soil samples with *Synechocystis* sp. and *Chorella sorokiniana* in the last weeks of the degradation experiment may be a clue that even the organic substances in soil have been faster degraded due to the higher microbial activity. Therefore, the soil carbon source was reduced compared to soil without biomass addition. This could mean that the overall determined cumulative biodegradation of the composite sample might be lower.

### Molecular weight as factor for the biodegradation

The molecular weight is one of the most important factors for biodegradation. The composites exhibits generally a much lower *M*_*w*_ than neat PHB, but with some variety depending on the composite. PHB with 100 mg/g *Synechocystis* sp. had the highest mass average molecular weight of all composites, whereas PHB with 100 mg/g *Cylindrospermum alatosporum* had the lowest. On the contrary, their cumulative biodegradation after 90 days lies within their respective standard deviations and reaches about 70 %, whereas pure PHB only biodegraded up to 18.7±1.1 %. Therefore, the mass average molecular weight *M*_*w*_ does not reflect the biodegradation behaviour of the composites. As consequence, a closer look at the molecular weight distribution and the binned molecular weights was necessary. From the distributions one could expect that the composites of PHB with *Chlorella* and *Cylindrospermum* would show the same biodegradation and that the composites with *Synechocystis* should degrade substantially slower (Fig. 2). It is more suitable to use the binned molecular weight fractions for discussion. The composites differ in the fractions below 80,000 g/mol and above 200,000 g/mol. Bonartsev et al. concluded that PHB chains can diffuse from the polymer sample below a molecular weight of 30,000 g/mol (Bonartsev et al. 2012). Since lower molecular weight PHB should be faster biodegradable than higher molecular weight PHB, the fraction of the polymer sample with a molecular weight below 80,000 g/mol should have the greatest impact on the CO_2_-evolution. In this range, neat PHB has 11.2 %, PHB-*Synechocystis* 26.1 %, PHB-*Chlorella* 36.7 % and PHB-*Cylindrospermum* 40.1 % of their sample weight. These differences are also not reflected in the biodegradation rates which are 18.7±1.1 %, 68.5±3.3 %, 71.3±2.1 % and 70.0±2.8 %, respectively. Therefore, neither the fraction of lower molecular weight nor the mass average molecular weight *M*_*w*_ seems to be a satisfying single explanation for the observed biodegradation rates. While molecular weight might be a partially explanation, this leads us to the assumption that there are other factors to consider.

### Decrease of molecular weight during preparation

Decrease of molecular weight of the PHB occurred during the compounding and injection moulding. In these steps, the polymer is subjected to temperatures around 180 ^∘^C. It is known, that PHB thermally degrades at temperatures above 160 ^∘^C within a short time frame (Kunioka and Doi 1990; Hoffmann et al. 1994). One study found a degradation of M_w_ from 1,028,000 g/mol to 41,800 g/mol after 30 min at 180 ^∘^C (Chen et al. 2013).

Another factor influencing the molecular weight decrease of PHB might be hydrolysis due to the water content of the sample. A study on PHB degradation with different clays a higher water content of the clay lead to stronger degradation of the PHB while also a catalyzing effect of one type of clay itself was suspected (Cabedo et al. 2009). Therefore, differences in water content of the PHB and biomass might contribute to the differences in molecular weight.

In other studies, even more rigorous drying procedures were used, but since the already spraydried materials were stored in a desiccator before use, most of the degradation and the differences between the composites can be attributed to the different processing temperatures and time durations of thermal treatment as a consequence of the rather manual processing steps.

### Influence of the crystalline fraction on biodegradation

Another important factor for the biodegradation rate is the crystalline fraction of the polymer phase. We assumed that the compounding with microbial biomass would lower the crystallinity of the PHB. Therefore, a less crystalline polymer would biodegrade faster (Spyros et al. 1997). In this study, a slight increase in crystallinity through addition of microbial biomass could be observed. Due to the small rise of the crystalline fraction, only a equally low negative influence on the biodegradation rates of the composites of PHB with microbial biomass can be assumed.

Other authors also found that biomass addition leads to changes in crystallinity. Barghini et al. observed a lower crystallinity for PHB composites when the marine seaweed *Ulva armoricana* was added (Barghini et al. 2010). On the contrary, Bulota et al. argued that the addition of algae particles leads to a higher crystallinity of polylactic acid samples due to an earlier onset of cold-crystallisation (Bulota and Budtova 2016). Changes of crystallinity by addition of biomass seems to be dependent on the used polymer and biomass source with the used microbial biomass here showing no effect on the crystallinity of PHB.

### Surface area and homogeneity of composites

A possibly enlarged surface area of the sample could only be indirectly analysed by visual observations. While SEM images did not reveal any obvious difference in surface area or roughness, non-homogeneously distributed huge aggregates such as those shown by *Synechocystis* sp. might lead to an increased surface roughness during the degradation process by leaving holes in the polymer matrix. Samples after biodegradation could not be examined, because particles could not be reliably separated from soil particles. Therefore, an influence of the microbial biomass on the surface area can not be suggested by this study. The raspberry-like aggregates of *Chlorella* were observed before by Zhang et al. They seem to be agglomerated *Chlorella*-cells, which forms a hollow sphere (Zhang et al. 2008).

### Influence of water uptake on biodegradation

As expected, the water uptake of PHB composites is higher than that of pure PHB. Microbial biomass allows the material to take up more water. According to Antheunis et al. a higher water content does not necessarily lead to a higher hydrolysis rate of the polymer (Antheunis et al. 2009). There might still be an influence on the mineralization by microorganisms or the water uptake may lead to a swelling of the material, which in turn might have triggered the enhanced biodegradation. However, the observed water uptakes and biodegradation rates do not support this idea, since the water uptake of PHB containing *Synechocystis* was much higher but the biodegradation rate was slightly lower than that of the other composites .

### Influence of biomass composition on biodegradation

As already argued, differences of substrate composition and available nutrients may influence the biodegradation by microorganisms. Since differences in molecular weight do not correspond to differences in biodegradation between composites, the composition remains as factor explaining the difference of biodegradation between pure PHB and its composites.

Since the composition (Table 3 and Table 4) does not vary much between the used biomass sources, the effect of single components in the microbial biomass can not be determined. However, the influence of biostimulation on biodegradation of compounds has been shown in several studies: An improved biodegradation on soils polluted with oil and hydrocarbons has been observed, if additional N and P sources are added to the soil (Martínez-Rivera and Cardona-Gallo 2021; Ruberto et al. 2009). A positive effect on the biodegradation of sodium benzoate by addition of NH_4_Cl or Mg_2_SO_4_ has been shown. The addition of phosphate exhibited a negative effect (Zaveri et al. 2021). Compost and nutrients addition has a positive effect on the biodegradation of petroleum (Salim et al. 2018). For ideal bioremediation, it is important that a suitable microbiome exists at the degradation location and that corresponding growth conditions with all its nutritional requirements are fulfilled (Tyagi et al. 2011). The enhancement of biodegradation seems to be a case of biostimulation by the addition of microbial biomass as a nutrient rich substrate to the PHB.

### Influence of microbial biomass on mechanical properties

Mechanical properties show a rise of the Young’s modulus and a decrease of tensile strength while elongation at break did not change much with the biomass addition. The biomass sources showed only a minor influence on mechanical properties with *Cylindrospermum alatosporum* having a lower Young’s modulus and a higher elongation at break than its corresponding composites. This observation fits well to the one made by Butola et al., who showed that the influence of different salt water macroalgae types on the mechanical properties is rather small. In their work, Young’s Modulus is increasing with algae content while strain at break and tensile strength decreases. This was also shown in another study with algal biomass after the extraction of the alginate (Bulota and Budtova 2015, 2016). Chiellini et al. observed similar results for another marine green algae (Chiellini et al. 2008). Zhang et al. found that the water content of *Chlorella* has an influence on the tensile strength of an polyvinyl chloride composite with *Chlorella*. The highest tensile strength can be achieved at a water content of 0.02 g/g and is greatly reduced at a higher water content (Zhang(b) et al. 2000). Zhu et al. argued that a certain water content of *Spirulina* is important for the processability of the composite (Zhu et al. 2017). Torres et al. showed similar results of lowered tensile strength and elongation at break for PBAT-composites with extracted microalgae biomass (Torres et al. 2015).

Variations in mechanical properties can also be attributed to differences in processing time (Hoffmann et al. 1994). Differences in water content were shown to influence mechanical properties, especially the elongation at break (Titone et al. 2021).

## Conclusion

In this study, the biodegradation and mechanical properties of PHB and PHB-composites with microbial biomass were investigated and the causes of the observed higher biodegradation rate of the composites were analysed. PHB-composites with green algae and cyanobacteria are biodegrading faster and to a higher extent than the individual components PHB and microbial biomass alone within 132 days.

We believe, that the most important factor for the improvement of biodegradation is a synergistic biostimulation effect, which was triggered by the combination of PHB with the microbial biomass and the occurring microfauna. Nevertheless, the drastic degradation of the molecular weight of the PHB polymer during processing of the samples, which is stronger for the composites than for PHB alone, is a second factor for the improved biodegradation. Since the difference in biodegradation between differten composites is rather small whereas there are bigger differences in their molecular weight, the effect of the degraded molecular weight seems to be rather small.

The crystallinity of the PHB increased slightly by the addition of the biomass. Water uptake and Young’s modulus of the composites are increased while the tensile strength is lowered. The use of microbial biomass for the preparation of PHB-composites was challenging due to the high susceptibility of molar mass to heat.

The observed increase of biodegradation rates and the possible synergistic biostimulation effect are an effective tool for tailoring the biodegradability and should be considered when investigating the biodegradation of composites.

This matter could be investigated further by testing the observed biostimulation effect in soil without microfauna, by addition of different compounds and nutrients instead of the microbial biomass to test their biostimulation effect and by transferring the concept to other biodegradable polymers like poly(lactic acid) or poly(butylene succinate).
